# Supplementary material for: A Catharanthus roseus Fe(II)/α-ketoglutarate-dependent dioxygenase catalyzes a redox-neutral reaction responsible for vindolinine biosynthesis
Source: Nat Commun. 2022 Jun 9;13:3335. doi: 10.1038/s41467-022-31100-1 (PMC9184523; doi:10.1038/s41467-022-31100-1)
Supplement: Supplementary file 1 — Supplementary Information [file 41467_2022_31100_MOESM1_ESM.pdf]

A *Catharanthus roseus* Fe(II)/α-ketoglutarate-dependent dioxygenase catalyzes a redox-neutral reaction responsible for vindolinine biosynthesis

Jasmine Ga May Eng[1], Mohammadamin Shahsavarani[2], Daniel Patrick Smith[1], Josef Hájíček[3] Vincenzo De Luca[4], and Yang Qu[1,2*]

[1] Department of Chemistry, University of New Brunswick, NB, Canada, [2] Department of Chemical Engineering, University of New Brunswick, NB, Canada, [3] Department of Chemistry, Charles University in Prague, Czech Republic, [4] Department of Biological Sciences, Brock University, ON, Canada

Supplementary table 1. Chemical shifts (^1H) of purified 19*S/R*-vindolinine and venalstonine in acetone *d*6 and $CDCl_3$ (400 MHz).

	Acetone *d*6		$CDCl_3$			
	19*S*-vindolinine	19*R*-vindolinine	19*S*-vindolinine	19*R*-vindolinine	Venalstonine	Venalstonine Reference*
NH	5.35 s	5.37 s	-	-	-	3.91 br s
2	-	-	-	-	-	
3	3.80 ddd 3.29 m	3.83 ddd 3.29 m	3.79 m 3.31 m	3.93 dd 3.30 ddd	3.47 m 3.47 m	3.47 m 3.47 m
5	3.43 ddd 3.29 m	3.43 ddd 3.29 m	3.47 ddd 3.31 m	3.47 m 3.43 dd	2.89 m 3.05 ddd	2.89 m 3.06 ddd
6	1.65 ddd 2.23 ddd	1.67 ddd 2.18 ddd	1.64 dd 2.21 dd	1.64 dd 2.17 ddd	?, 2.57 ddd	1.35 m 2.58 ddd
7	-	-	-	-	-	-
8	-	-	-	-	-	-
9	6.99 d	7.07 d	7.26 d	7.25 d	7.08 d	7.10 d
10	6.67 dt	6.70 dt	6.87 t	6.86 dt	6.75dt	6.75 dt
11	6.95 dt	6.96 dt	7.07 dt	7.07 dt	7.02 dt	7.03 dt
12	6.62 d	6.64 d	6.80 d	6.79 d	6.70 d	6.70 d
13	-	-	-	-	-	-
14	5.75 ddd	5.75 ddd	5.79 ddd	5.77 ddd	5.72 dt	5.73 dt
15	6.16 dd	6.13 dd	6.21 br d	6.14 dd	5.51 dt	5.53 dt
16	3.06 dd	3.10 dd	3.04 dd	3.04 dd	2.89 m	2.89 m
17	2.47 dd 1.70 dd	2.44 ddd 1.77 ddd	1.78 dd 2.52 dd	1.82 ddd 2.47 ddd	?, 1.90 ddd	1.24 ddd 1.88 ddd
18	0.56 d	0.99 d	0.59 d	0.95 d	1.63 m 2.68 ddd	1.64 m 2.68 ddd
19	2.15 q	1.99 q	2.11 q	2.08 q	?, 1.63 m	1.35 m 1.64 m
20	-	-	-	-		-
21	3.30 s	3.34 s	3.49 s	3.49 s	2.74s	2.74 s
COOCH3	3.64 s	3.64 s	3.72 s	3.70 s	3.74 s	3.74 s

* The reference spectra were kindly provided by Prof. Kam Toh Seok at the University of Malaya, Malaysia [1,2].

Supplementary table 2. The major alkaloids contents in leaf and flower tissues of two *Catharanthus roseus* varieties Little Delicata and Pacifica White.

		Post-stemmadenine alkaloids (mg/g fresh plant material)					Other major alkaloids (mg/g fresh plant material)		
		Total Vindoline& Vindorosine	Catharanthine	Vindolinine (total epimers)	19*S*-vindolinine	19*R*-vindolinine	Serpentine	Perivine	ajmalicine
cv. Little Delicata (this study)	Young leaf	1.631±0.589	0.641±0.243	**0.256±0.077**	0.153±0.053	0.103±0.026	0.157±0.060	0.070±0.026	0.042±0.036
	Flower	0.093±0.035	0.077±0.032	**0.102±0.035**	0.068±0.024	0.034±0.011	0.045±0.021	0.008±0.001	0.022±0.015
cv. Pacifica white (popular variety)	Young leaf	1.495±0.431	0.470±0.183	**0.147±0.045**	0.092±0.021	0.054±0.024	0.127±0.016	0.032±0.010	0.046±0.033
	Flower	0.198±0.017	0.051±0.010	**0.098±0.024**	0.058±0.013	0.041±0.012	0.029±0.006	0.004±0.002	0.011±0.006

Supplementary table 3. Primer list.

Primer #	Primer name	Sequence
1	attb-VNS-F	GGGGACAAGTTTGTACAAAAAAGCAGGCTTCATGGCAGGATCAGTACTTA
2	attb-VNS-stop-R	GGGGACCACTTTGTACAAGAAAGCTGGGTCCTATAATTTCATAGCATCAAGTGTAA
3	attb-VNS-R	GGGGACCACTTTGTACAAGAAAGCTGGGTCTAATTTCATAGCATCAAGTGTAA
4	VNS-BamHI-F	CGCAGGATCCGATGGCAGGATCAGTACTTACC
5	VNS-SalI-R	CGCGGTCGACCTATAATTTCATAGCATCAAGTGTAA
6	VIGS-VNS-F	ATAGGAATTCGGAGCTCAAGCTAAGGATTGGACA
7	VIGS-VNS-R	ATACGAATTCTTAGGGTCATTACCCCTCCATCTG
8	qPCR-60S-F	TCTTAGTTGGAATGTTCAGCACCTG
9	qPCR-60S-R	CAAGGTTGGAGCCCCTGCTCGTGTT
10	qPCR-VNS-F	CCCGAACTCGTATTGGGGTTGAAA
11	qPCR-VNS-R	GCCAGGACGTGGATGAACTTGAT
12	qPCR-GO-F	TATGGCCCGAACCAGAGAAGTTTG
13	qPCR-GO-R	GACATCCTCTTCTACCTGTCCCAA
14	qPCR-IS-F	GTTATGCTGATGCTGTTGATGC
15	qPCR-IS-R	CCATTTCAATCCCAAATTCCTCTG
16	qPCR-D4H-F	GGGTTTCGCCAAGATTGTACGGA
17	qPCR-D4H-R	CGTCAAAGCGTTTGGCAAATCG

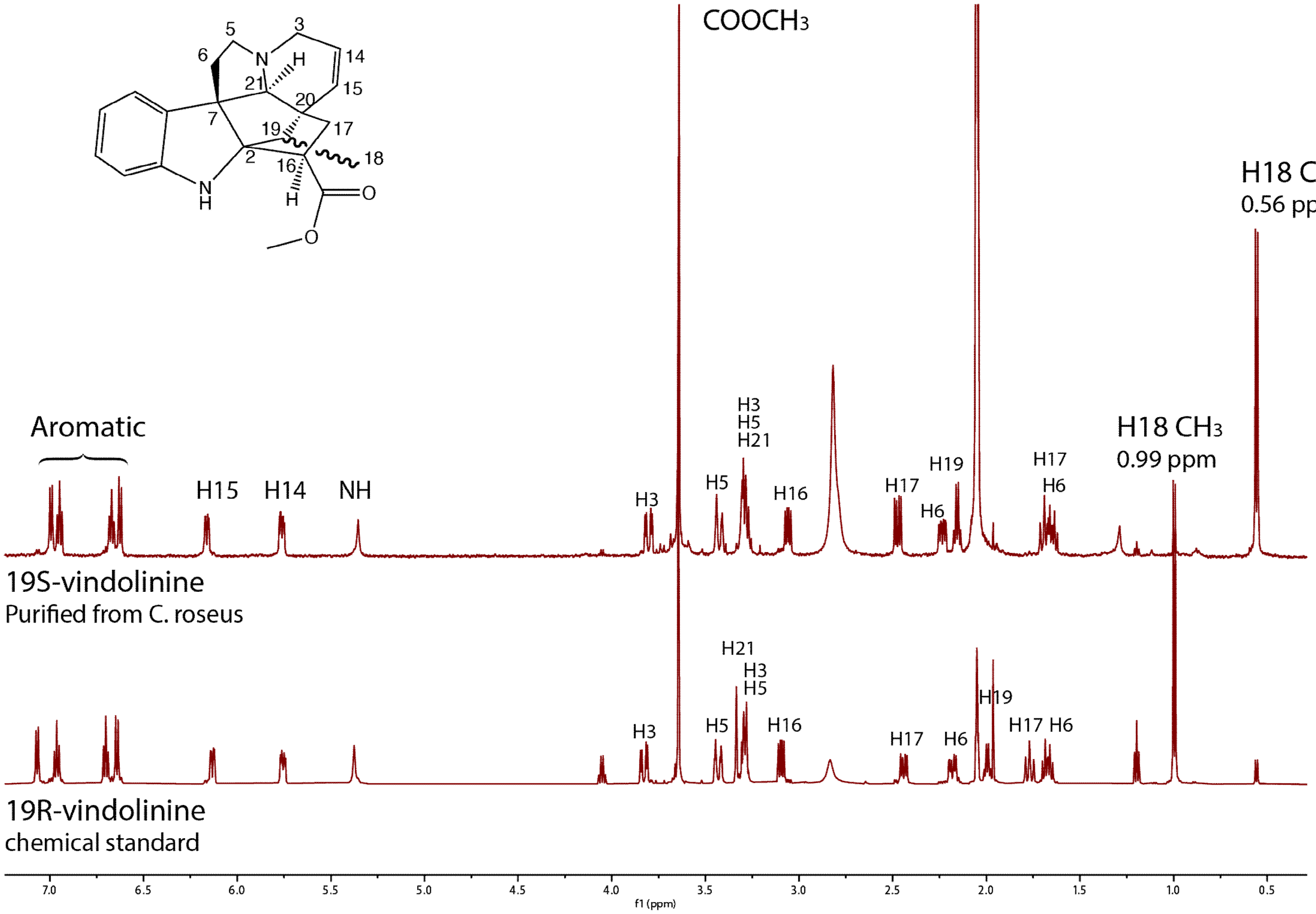

Supplementary Figure 1. NMR (^{1}H) spectra of 19*S*-vindolinine (top) and 19*R*-vindolinine (bottom) in acetone-*d*6. 19*R*-vindolinine (vindolinine) was supplied by Sigma Aldrich, whereas 19*S*-vindolinine (epivindolinine) was purified from *Catharanthus roseus* total leaf alkaloids. The NMR spectra of both epimers are highly similar, and consistent with the NMR spectra reported in literature [3,4]. The most notable difference between the two epimers is the chemical shifts of the C18 methyl (0.56 ppm for 19*S* stereochemistry and 0.99 ppm for 19*R* stereochemistry), followed by those of the C19 methine (2.15 ppm for 19*S* stereochemistry and 1.99 ppm for 19*R* stereochemistry). The chemical shifts are listed in Supplementary table 1.

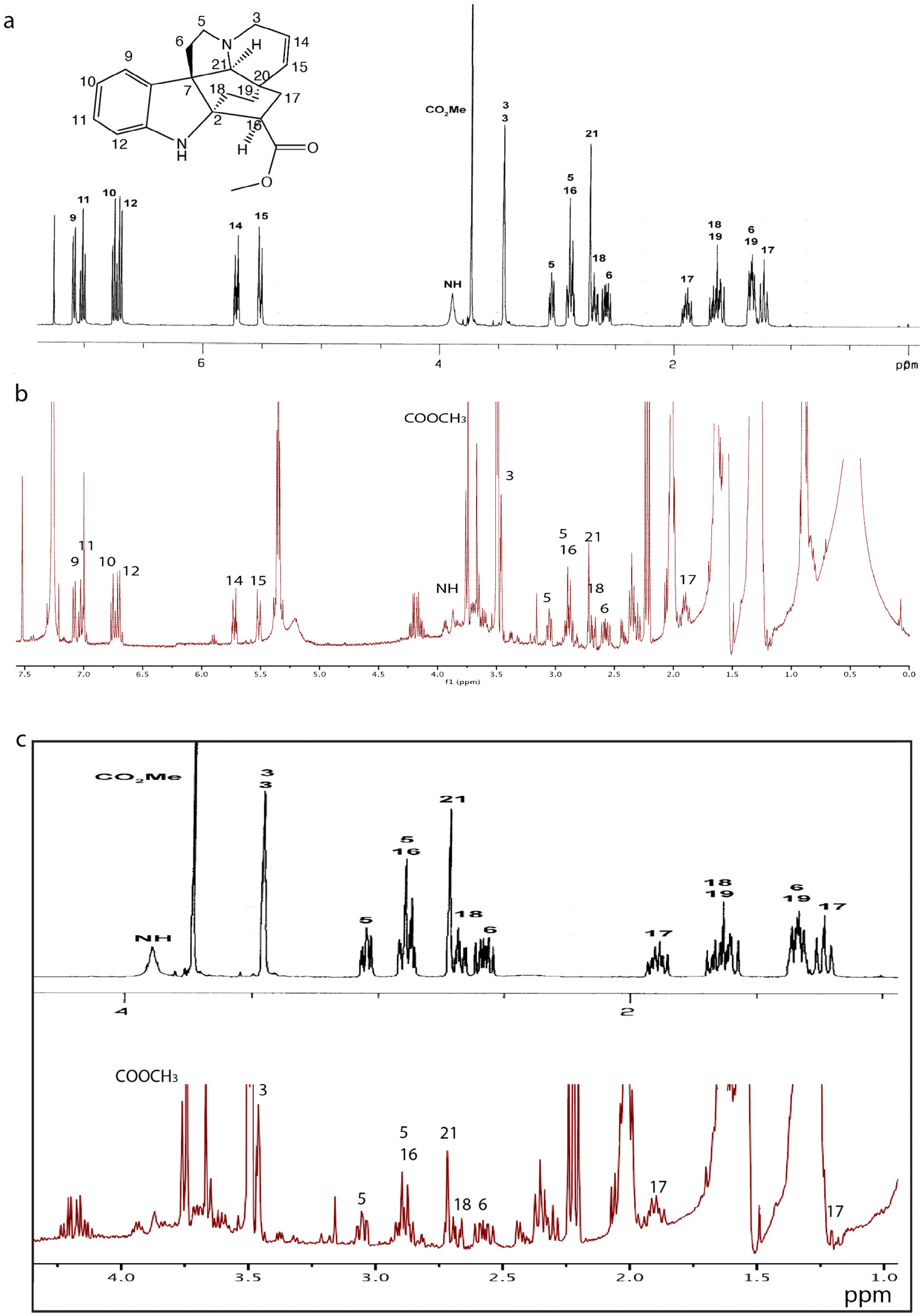

Supplementary Figure 2. NMR (^{1}H) spectra of reference venalstonine (**a**) and venalstonine purified from *Catharanthus roseus* total leaf alkaloids in this study (**b**) in $CDCl_3$. The reference venalstonine NMR spectra were kindly provided by Prof. Kam Toh Seok at the University of Malaya, Malaysia [1,2]. The semi-purified venalstonine showed identical chemical shifts with the reference venalstonine. The chemical shifts are listed in Supplementary table 1. (**c**) shows the chemical shifts comparison in the range of 1-4 ppm between the reference venalstonbine ^{1}H spectra and those of venalstnonine purified from this study.

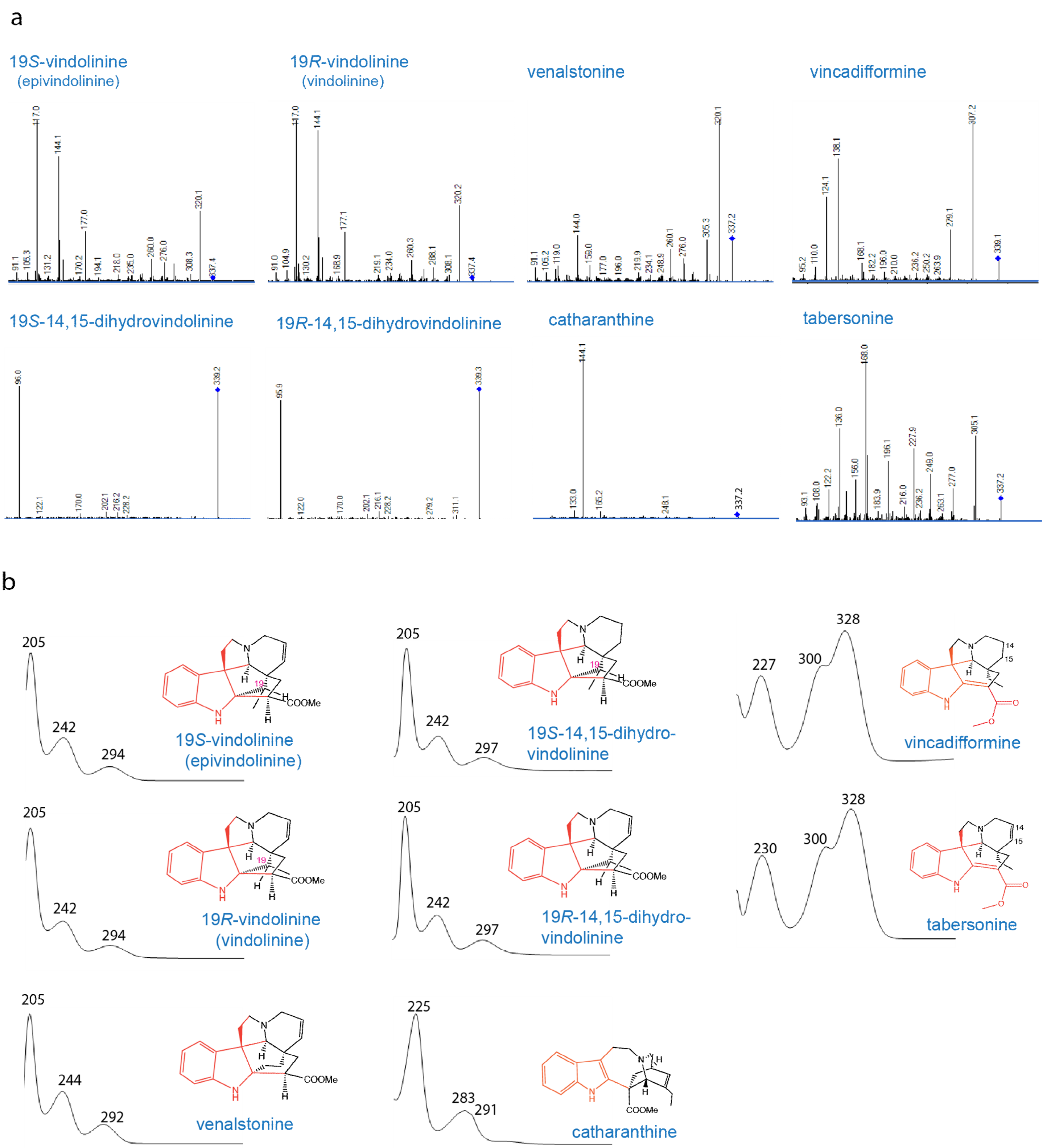

Supplementary figure 3. The electrical spray ionization (ESI) MS/MS spectra (**a**) and UV absorption profiles (**b**) of the MIAs in this study. Both the 19*S/R*-vindolinines and venalstonine showed a signature daughter ion *m/z* 320, which indicates the loss of O^- (16 amu). They all have the signature indole daughter ion *m/z* 144 ($C_{10}H_{10}N^+$), which is also evident in catharanthine. The UV absorption spectra (205, 243, 294 nm) are typical for dihydroindole chromophore.

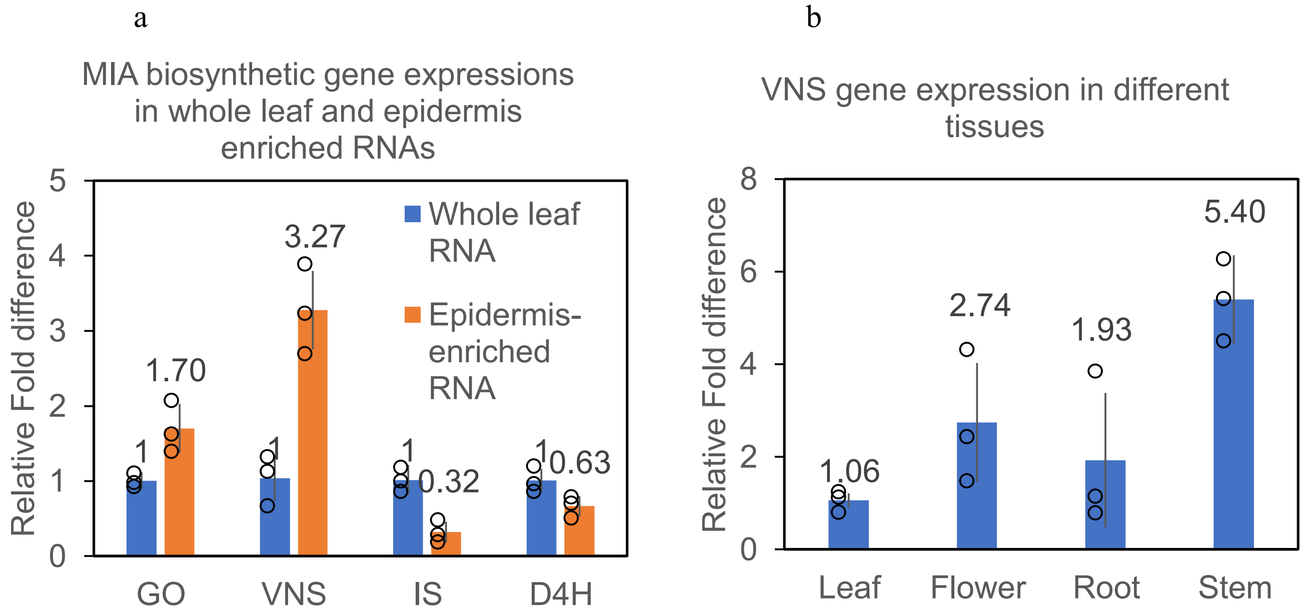

Supplementary figure 4. The transcripts of VNS were higher in the epidermis-enriched leaf total RNAs compared to the whole leaf total RNA (**a**). The expression of four MIA biosynthetic genes were tested by qRT-PCR in epidermis-enriched leaf total RNA prepared using carborundum abrasion and in whole *C. roseus* leaf. The epidermal MIA gene GO also showed higher transcripts in epidermis-enriched leaf total RNA, while the internal phloem associated parenchyma cell (IPAP)-localized iridoid synthase (IS) and laticifer/idioblast-localized deacetoxyvindoline 4-hydroxylase (D4H) showed reduced expression in these RNA sets. Three independently prepared leaf epidermis-enriched RNA extracts and three independently prepared whole leaf tissue RNA extracts were used in these studies. The unpaired two-tailed Student's *t*-tests were used to evaluate the differences between the gene expression in the whole leaf and epidermis-enriched leaf RNA for reach gene. The p-values for each gene are: GO, 0.0276; VNS, 0.0050; IS, 0.0055; D4H, 0.0577. (**b**) VNS expression in four *C. roseus* tissues: leaf, flower, root, and stem by qRT-PCR. For each tissue type, three independently prepared RNA extracts were used. The gene 16S ribosome RNA was used to normalize each RNA sets. The error bars indicate standard deviations from three biological replicates and three qRT-PCR technical replicates.

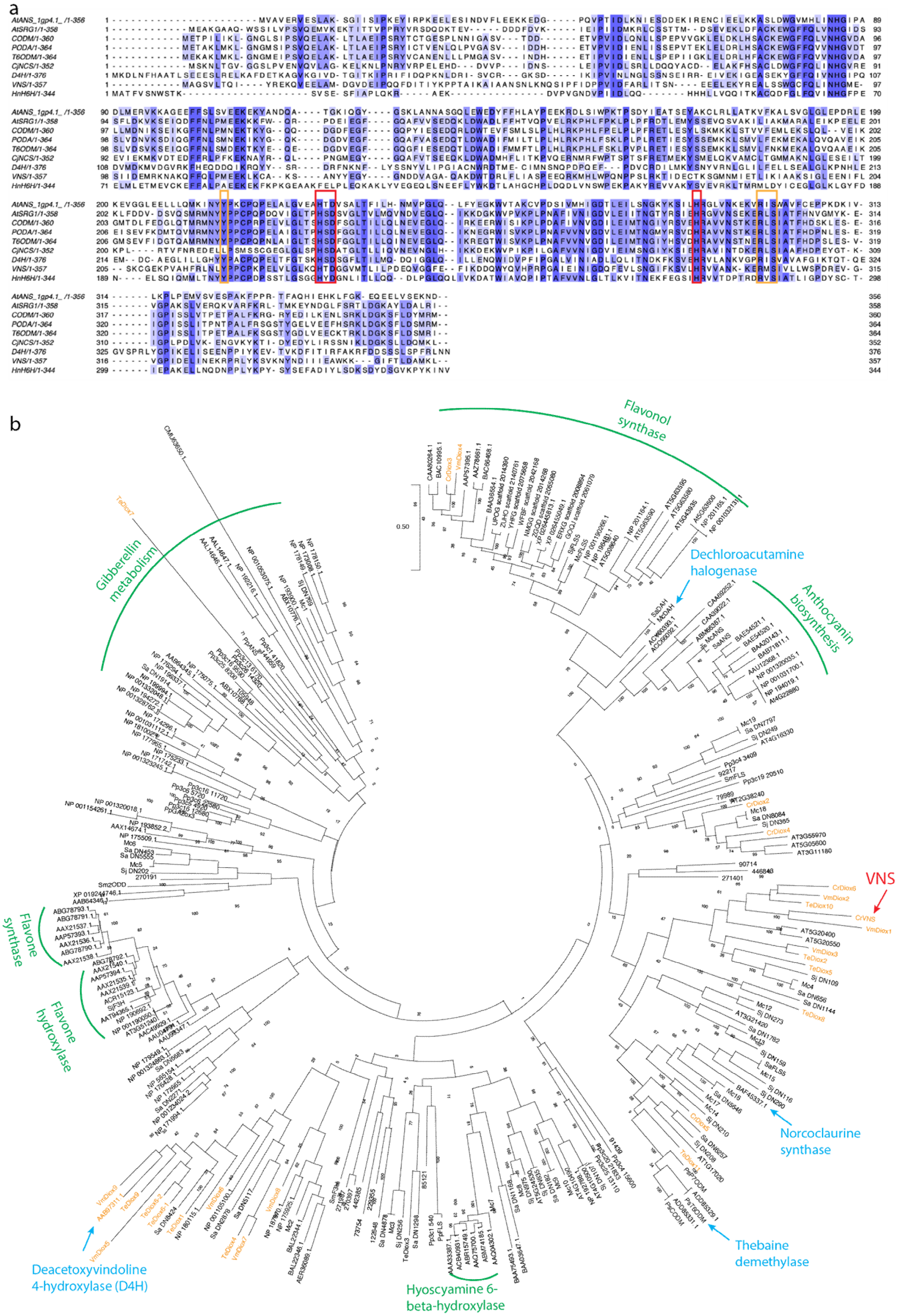
a
AtANS_1gp4.1_/1-356
AtSRG1/1-358
CODM/1-360
PODA/1-364
T6ODM/1-364
CjNCS/1-352
D4H/1-376
VNS/1-357
HnH6H/1-344
b
Flavonol synthase
Dechloroacutamine halogenase
Anthocyanin biosynthesis
Gibberellin metabolism
Flavone synthase
Flavone hydroxylase
VNS
CrVNS
Norcoclaurine synthase
Thebaine demethylase
Hyoscyamine 6-beta-hydroxylase
Deacetoxyvindoline 4-hydroxylase (D4H)
0.50

Supplementary figure 5. Amino acid sequence alignment of vindolinine synthase (VNS) with other plant Fe/2OG-dioxygnases (**a**) and the phylogenetic tree of VNS with other Fe/2OG-dioxygnases (**b**). The red boxes indicate the conserved HXDXnH catalytic triad required for coordinating Fe(II) and the orange boxes indicate the YXnRXS motif involved in α-ketoglutarate binding. The protein sequences were retrieved from www.phytometasyn.ca and three reference articles [5–7]. The evolutionary history was inferred by using the Maximum Likelihood method and Poisson correction model [8]. The bootstrap consensus tree inferred from 100 replicates is taken to represent the evolutionary history of the taxa analyzed [9]. Branches corresponding to partitions reproduced in less than 50% bootstrap replicates are collapsed. Initial tree(s) for the heuristic search were obtained automatically by applying Neighbor-Join and BioNJ algorithms to a matrix of pairwise distances estimated using the Poisson model, and then selecting the topology with superior log likelihood value. This analysis involved 261 amino acid sequences. Evolutionary analyses were conducted in MEGA X [10,11]. AtANS: *Arabidopsis thaliana* anthocyanin synthase; AtSRG1: *Arabidopsis thaliana* senescence-related gene-1; PsCODM: *Papaver somniferum* codeine 3-O-demethylase; PsT6ODM: *Papaver somniferum* thebaine 6-O-demethylase; PODA: protopine O,O-demethylenase; CjNCS: *Coptis japonica* norcoclaurine synthase; HnH6H: *Hyoscyamus niger* hyoscyamine 6β-hydroxylase; CrD4H: *Catharanthus roseus* deacetoxyvindoline 4-hydroxylase; McDAH: *Menispermum canadense* dechloroacutamine halogenase. The dioxygenases from three Apocynaceae species (*Catharanthus roseus, Vinca minor, and Tabernaemontana elegans*) used in this phylogeny analysis are labeled in orange colour.

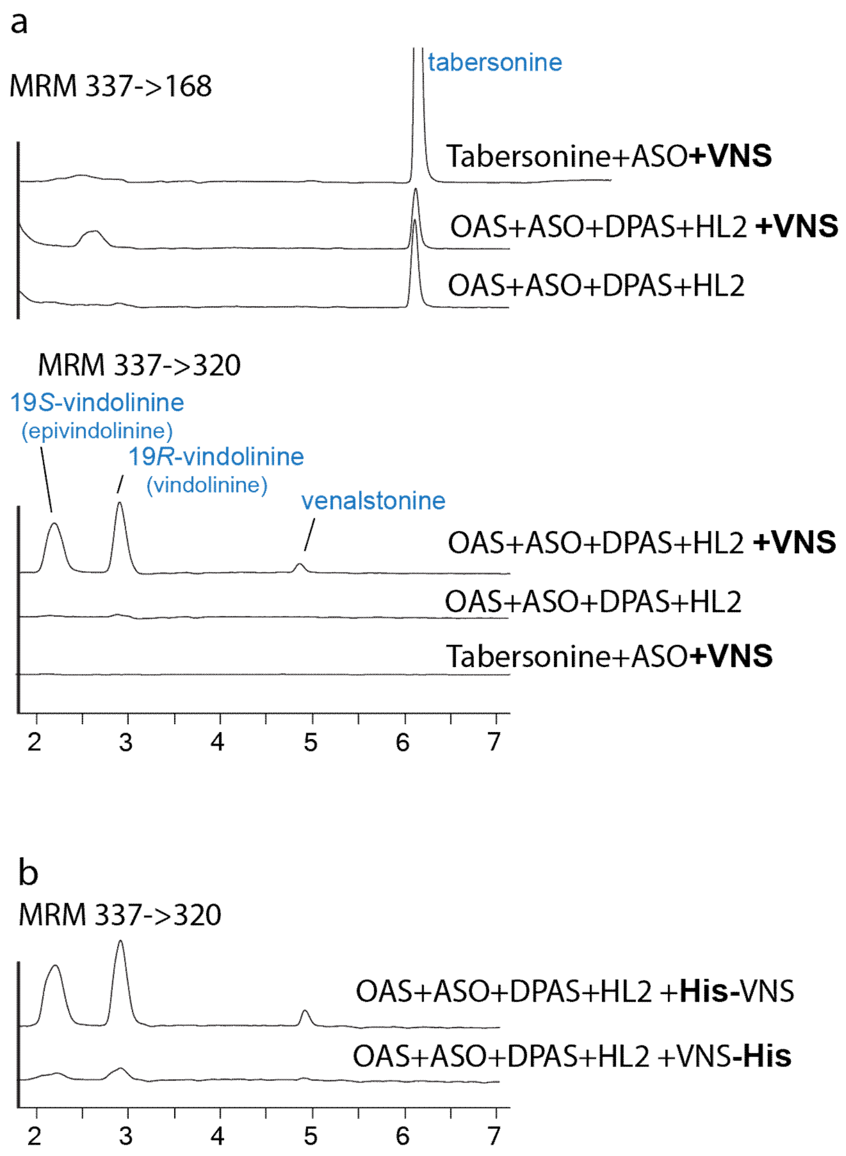

Supplementary figure 6. The formation of 19*S/R*-vindolinines and venalstonine by feeding *O*-acetylstemmadenine (OAS) or tabersonine substrates to tobacco leaves transiently co-expressing the enzymes: *O*-acetylstemmadenine oxidase (ASO), dihydroprecondylocarpine acetate synthase (DPAS), hydrolase 2 (HL2), and vindolinine synthase (VNS). The LC-MS/MS traces show the multiple reaction monitoring (MRM) chromatograms with the transitions of *m/z* 337->168 for tabersonine, *m/z* 337->320 for 19*S/R* vindolinines and venalstonine. (**a**) Both 19*S/R*-vindolinines and venalstonine were formed in the feeding assay with OAS substrate and ASO, DPAS, HL2 and VNS. Omission of HL2 resulted in the disappearance of these three MIAs while tabersonine could still be formed. (**b**) The C-terminal 6x His-tag on VNS almost abolished VNS activity, comparing to normal vindolinine forming activity when the His-tag is located at the N-terminal of VNS.

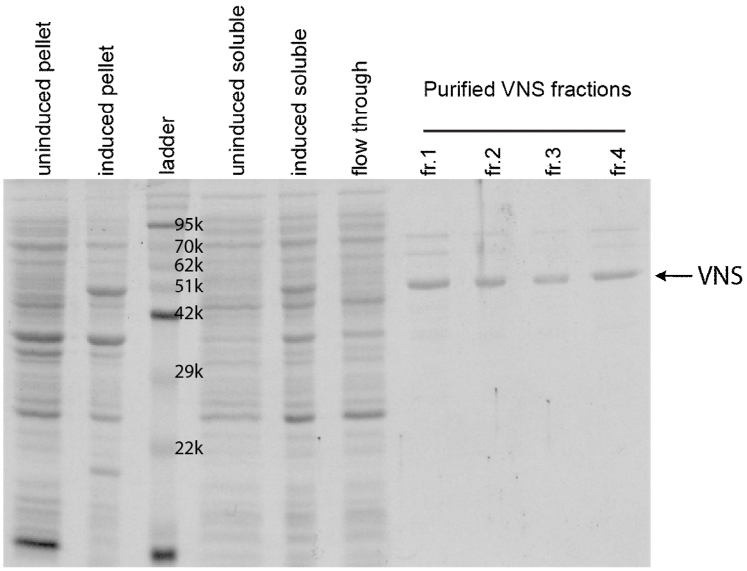

Supplementary figure 7. Purification of 6X-His-tag vindolinine synthase (VNS) from *E. coli*. The induction of VNS soluble protein was evident when compared to the non-induced control when the proteins were separated on SDS-PAGE. The gel image was generated from a single purification experiment without replicates.

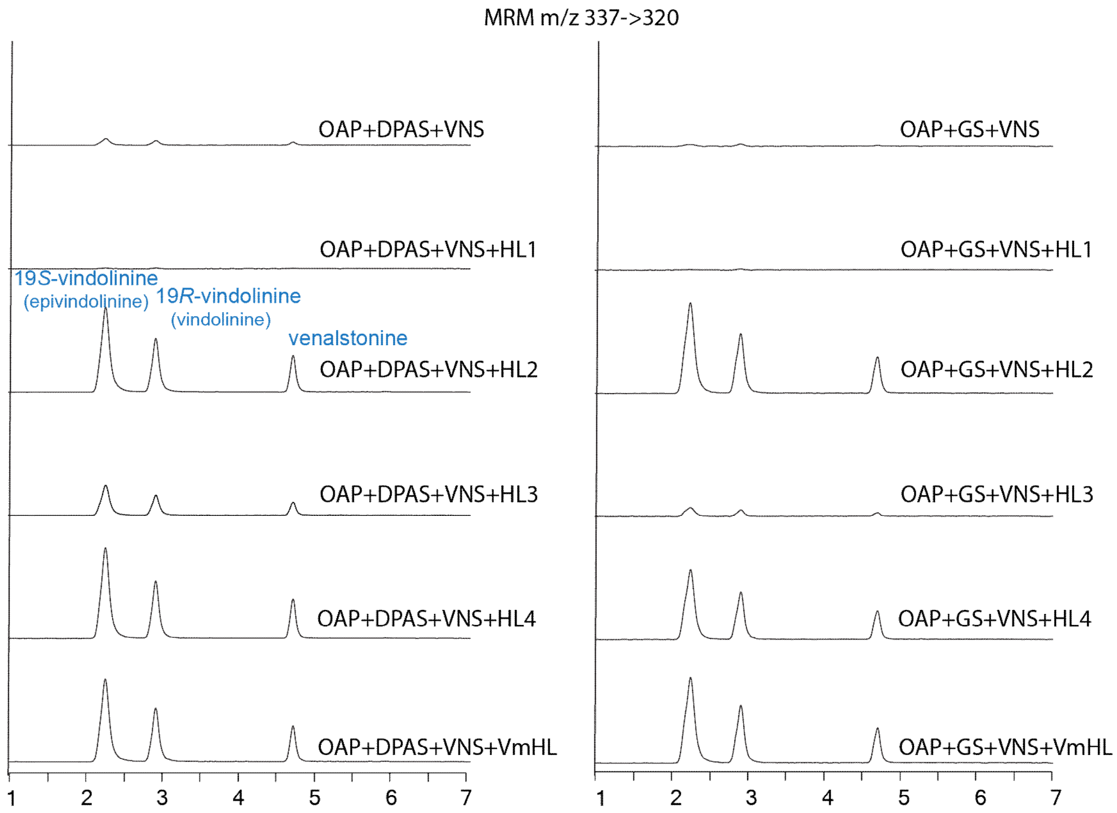

Supplementary figure 8. The biosynthesis of *19S/R*-vindolinines and venalstonine requires the reduction of *O*-acetylprecondylocarpine (OAP) by dihydroprecondylocarpine acetate synthase (DPAS) (left panel) or geissoschizine synthase (GS) (right panel) and the cyclization by a hydrolase. Recombinant DPAS, GS, VNS, HL1-4 and VmHL (*Vinca minor* hydrolase) were affinity-purified from *E. coli* and assayed *in vitro* with the substrate *O*-acetylprecondylocarpine (OAP). The product formation was analyzed by LC-MS/MS MRM *m/z* 337->320. Both DPAS and GS could be used for OAP reduction to dehydrosecodine, while HL2, 4 and VmHL could all cyclize the VNS-produced radical to the vindolinines and venalstonine. HL3 comparatively showed reduced activity, whereas HL1 that is responsible for iboga MIA formation showed no activity for vindoline formation.

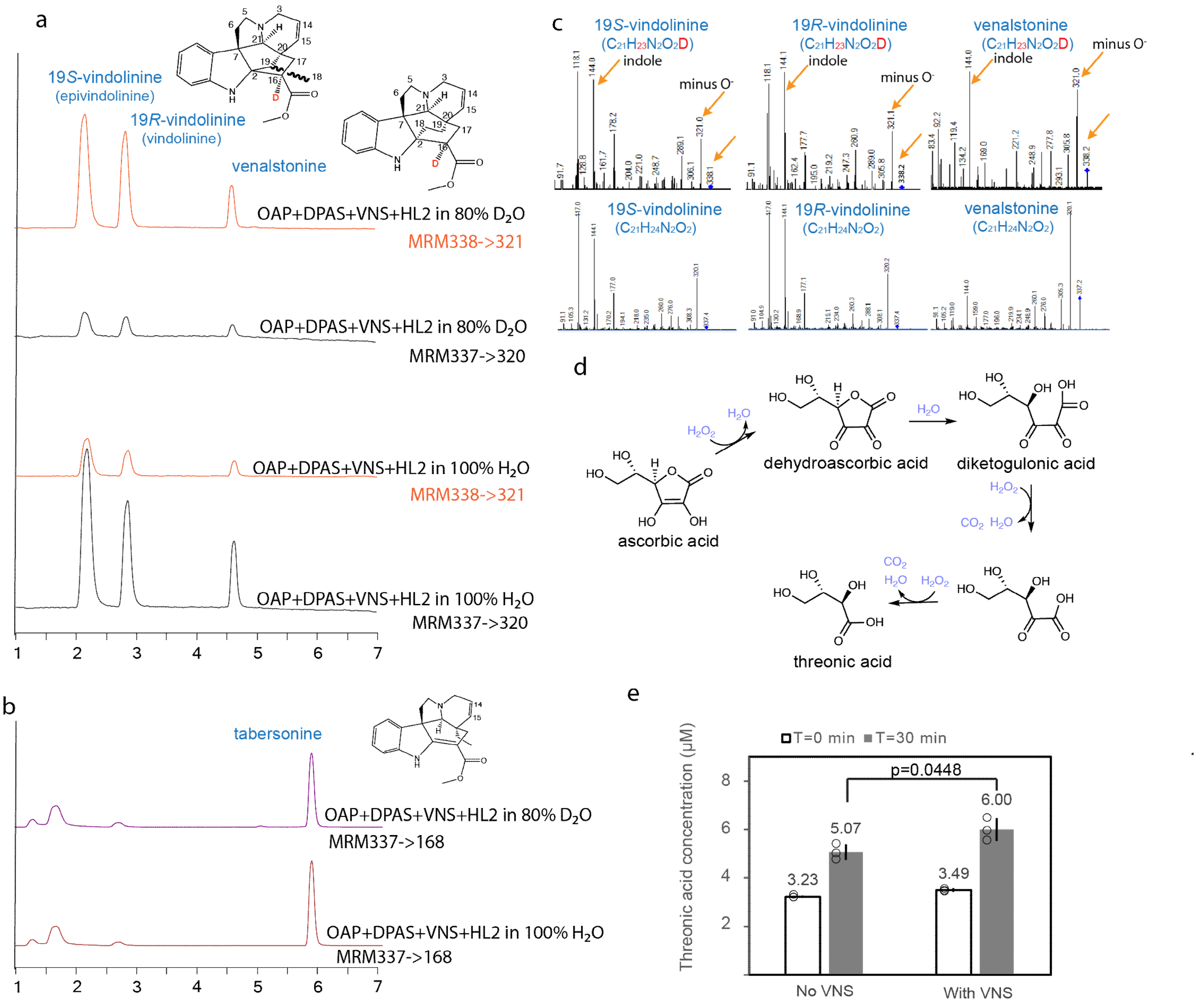

Supplementary figure 9. The biosynthesis of *19S/R*-vindolinines and venalstonine involves the incorporation of a hydrogen from water. Both *19S/R*-vindolinines and venalstonine are isomers of $C_{21}H_{24}N_2O_2$ (m/z 337). Reacting the substrate O-acetylprecondylocarpine (OAP) with dihydroprecondylocarpine acetate synthase (DPAS), vindolinine synthase (VNS), and hydrolase 2 (HL2) in H_2O forms the expected alkaloids (MRM *m/z* 337->320). Small amounts of MRM *m/z* 338->321 could also be detected by LC-MS/MS, mostly due to the low MS resolution of triple quadruple (QQQ) MS instrument used in this study. Replacing 80% of the reaction with deuterium oxide (D_2O) clearly resulted in heavier *19S/R*-vindolinines and venalstonine, as the masses of the majority of the products all increased by 1 amu (**a**). MS spectra of the deuterated vindolinines and venalstonine compared to the non-deuterated versions (**b**). Both the parental ion (*m/z* 338) and the signature daughter ion (*m/z* 321) were increased by 1 amu from *m/z* 337 and *m/z* 321 when the reactions were in D_2O. The indole fragment ($C_{10}H_{10}N^+$) *m/z* 144 however stayed non-changed, since the incorporation of deuterium is only on C16. The amounts of tabersonine formed in both H_2O and D_2O solution remained the same (**c**), suggesting its formation does not involve water. (**d**) The degradation pathway for ascorbic acid when oxidized by H_2O_2 [11]. (**e**) A statistically significant (p=0.0448) increase of threonate was observed in OAP+DPAS+VNS+HL2 reactions compared to the same reactions without VNS. Threonate concentrations were measured at T=0 min and 30 min, when all OAP has been converted. The data is mean value of three replicates at each time point, and unpaired two tailed Student's *t*-test was used for statistical analyses. Error bards indicate standard deviation.

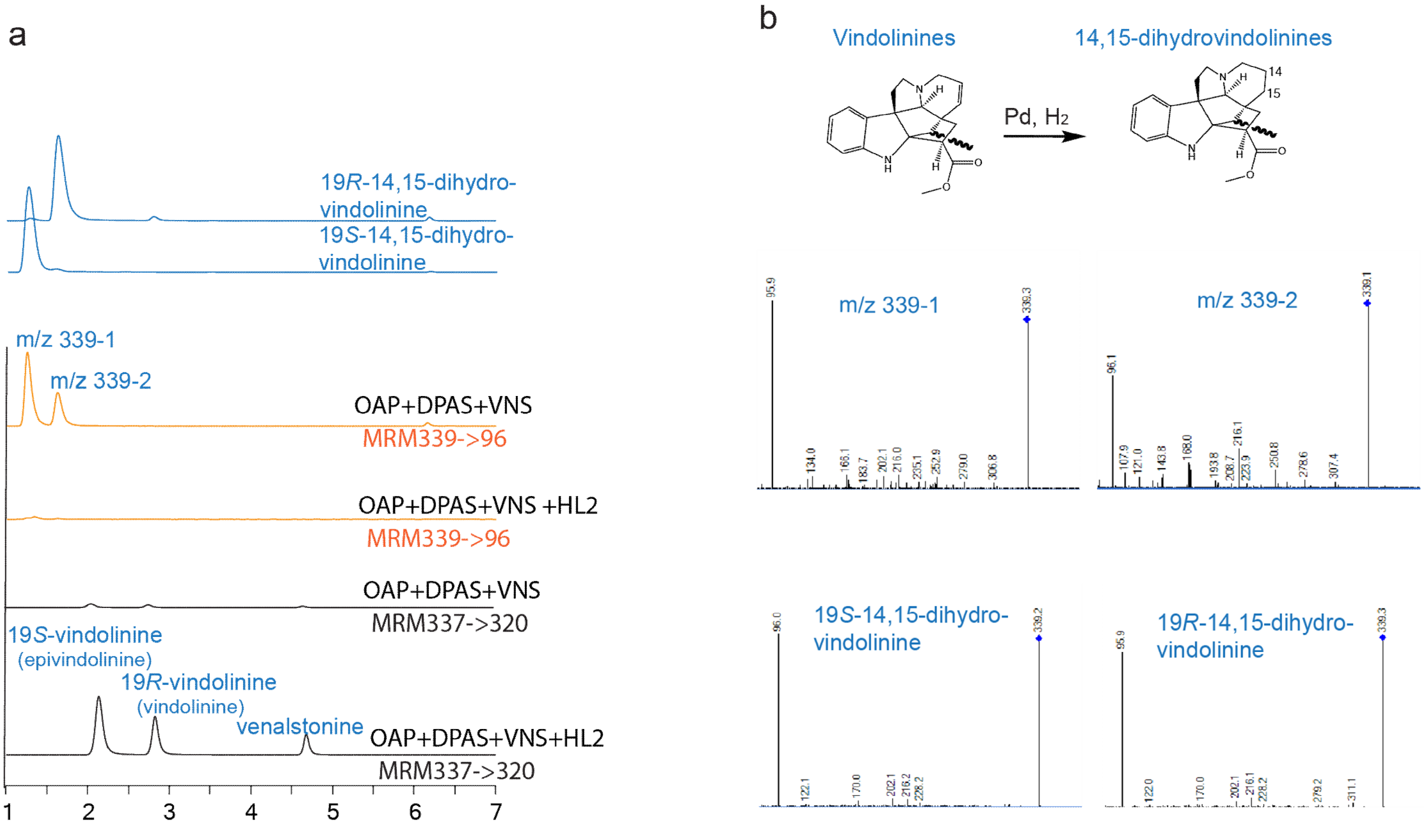

Supplementary figure 10. The *19S/R*-14,15-dihydrovindolinines are formed instead in the absence of hydrolase 2 (HL2) when *O*-acetylprecondylocarpine (OAP) is reacted with only dihydroprecondylocarpine acetate synthase (DPAS) and vindolinine synthase (VNS). With DPAS, VNS, HL2, vindolinines and venalstonine (*m/z* 337) were formed from OAP as expected. Removing HL2 in the reaction resulted in the disappearance of these alkaloids and the appearance of two further reduced, isomeric alkaloids with *m/z* value of 339 (**a**). Chemically reduction of 14,15-double bonds in the vindolinines by palladium and H_2 gas at room temperature formed the 14,15-dihydrovindolinines. The MS spectra of chemical synthesized 14,15-dihydrovindolinines were identical with the two *m/z* 339 alkaloids in the enzymatic reactions (**b**). The liquid chromatography retention times also matched between the chemically and enzymatically formed alkaloids.

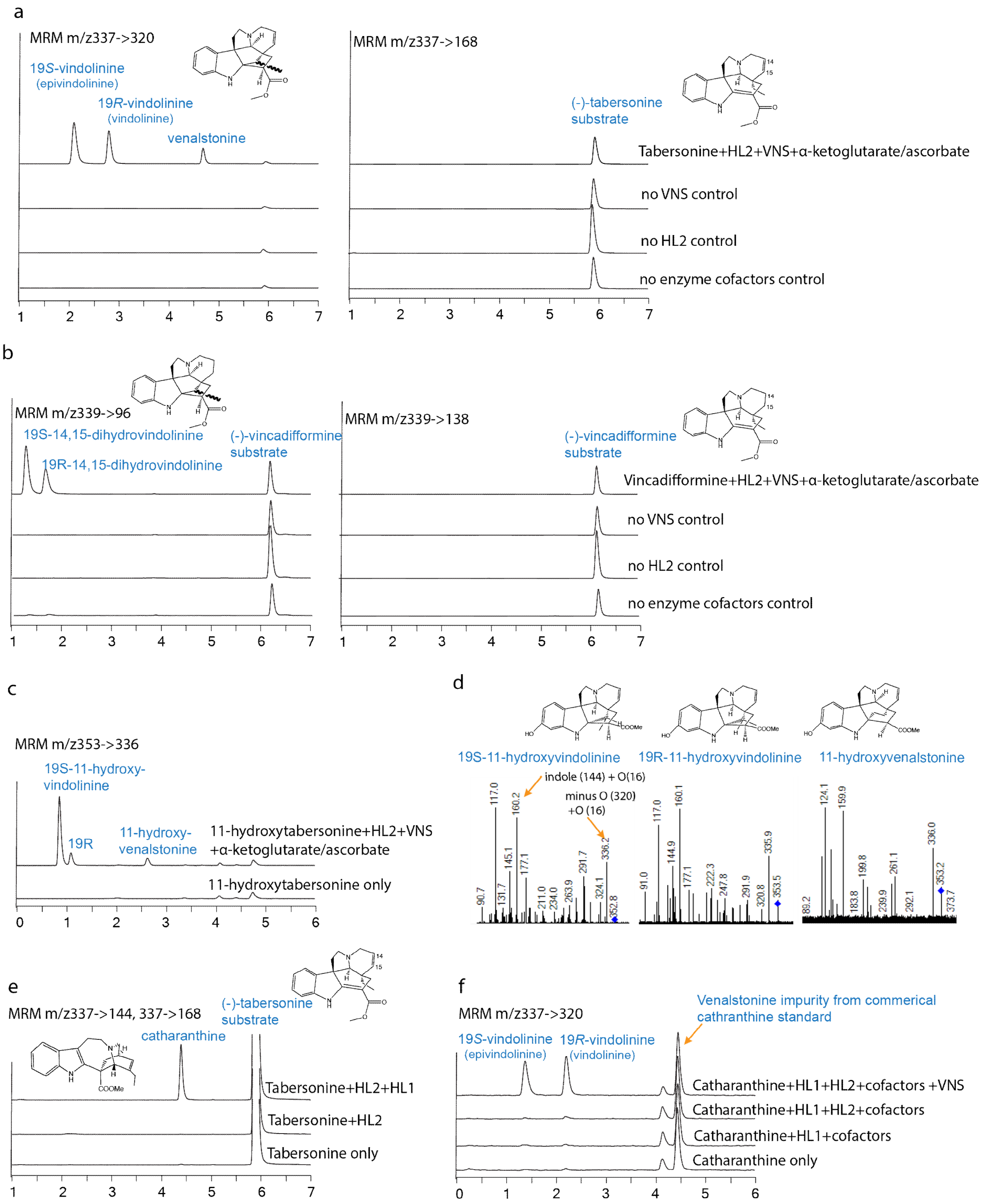

Supplementary figure 11. Hydrolase 2 (HL2) catalyzes a reverse reaction to generate dehydrosecodine, which is taken by vindolinine synthase (VNS) and HL2 in a forward reaction to generate the vindolinines and venalstonine (**a**). Removing VNS, HL2 or the VNS co-factors α-ketoglutarate/ascorbate all resulted in the abolishment of vindolinine formation. Using the 14,15-dihydrotabersonine (vincadifformine), the same results were obtained with the formation of 14,15-dihydrovindolinines (**b**). HL2 and VNS could also accept the indole hydroxylated alkaloid 11-hydroxytabersonine as a substrate and produce the 11-hydroxyvindolinines and 11-hydroxyvenalstonine in the same reverse reaction, with a strong preference for the 19*S*-stereochemistry (**c**). The MS spectra of both 11-hydroxyvindolinines and 11-hydroxyvenalstonine all showed expected daughter ions including the minus O^- ion *m/z* 336 (320+16), and the hydroxyindole ion *m/z* 160 (144+16) (**d**). Tabersonine is converted to dehydrosecodine by the reverse-cycloaddition activity of HL2, which is further cyclized by HL1 to form catharanthine (**E**). Trace amounts of vindolinine epimers were formed by reacting catharanthine with HL1, HL2, and VNS (**F**).

Supplementary References

1. Subramaniam, G. *et al.* Biologically Active Aspidofractinine Alkaloids from *Kopsia singapurensis*. *J Nat Prod* 71, 53–57 (2007).

2. Kam, T.-S. & Choo, Y.-M. Venalstonine and dioxokopsan derivatives from *Kopsia fruticosa*. *Phytochemistry* 65, 2119–2122 (2004).

3. Durham, L. J., Shoolery, J. N. & Djerassi, C. Reinvestigation of the Proton Resonance Spectrum of Vindolinine at 300 MHz. *Proc National Acad Sci* 71, 3797–3799 (1974).

4. Atta-Ur-Rahman, Malik, S., & Albert, K. Structural Studies on Vindolinine. *Z. Naturforsch.* 41, 386–392 (1986).

5. Hagel, J. M. & Facchini, P. J. Dioxygenases catalyze the *O*-demethylation steps of morphine biosynthesis in opium poppy. *Nature chemical biology* 6, 273–275 (2010).

6. Kawai, Y., Ono, E. & Mizutani, M. Evolution and diversity of the 2–oxoglutarate-dependent dioxygenase superfamily in plants. *Plant J* 78, 328–343 (2014).

7. Kim, C. Y. *et al.* The chloroalkaloid (−)-acutumine is biosynthesized via a Fe(II)- and 2-oxoglutarate-dependent halogenase in Menispermaceae plants. *Nat Commun* 11, 1867 (2020).

8. Zuckerkandl E. and Pauling L. (1965). Evolutionary divergence and convergence in proteins. Edited in Evolving Genes and Proteins by V. Bryson and H.J. Vogel, pp. 97-166. Academic Press, New York.

9. Felsenstein J. (1985). Confidence limits on phylogenies: An approach using the bootstrap. Evolution 39:783-791.

10. Kumar S., Stecher G., Li M., Knyaz C., and Tamura K. (2018). MEGA X: Molecular Evolutionary Genetics Analysis across computing platforms. Molecular Biology and Evolution 35:1547-1549.

11. Stecher G., Tamura K., and Kumar S. (2020). Molecular Evolutionary Genetics Analysis (MEGA) for macOS. Molecular Biology and Evolution (https://doi.org/10.1093/molbev/msz312).

12. Dewhist, R. A. & Fry S. C. The oxidation of dehydroascorbic acid and 2,3-diketogulonate by distinct reactive oxygen species. *Biochem J* **475**, 3451-3470 (2018)
